# First-Principles Study of Electronic Properties of Substitutionally Doped Monolayer SnP_3_

**DOI:** 10.3390/ma15072462

**Published:** 2022-03-27

**Authors:** Ningxia Zhang, Xiaodan Li, Shihao Ruan, Xiong Chen, Shenghao Li, Taotao Hu

**Affiliations:** 1 College of Science, University of Shanghai for Science and Technology, Shanghai 200093, China; 182282021@st.usst.edu.cn (N.Z.); 202232248@st.usst.edu.cn (S.R.); 202232244@st.usst.edu.cn (X.C.); 202232236@st.usst.edu.cn (S.L.); 2 School of Physics, Northeast Normal University, Changchun 130024, China; hutt262@nenu.edu.cn

**Keywords:** first-principles, two-dimensional materials, substitutional doping, monolayer SnP_3_

## Abstract

SnP_3_ has a great prospect in electronic and thermoelectric device applications due to its moderate band gap, high carrier mobility, absorption coefficients, and dynamical and chemical stability. Doping in two-dimensional semiconductors is likely to display various anomalous behaviors when compared to doping in bulk semiconductors due to the significant electron confinement effect. By introducing foreign atoms from group III to VI, we can successfully modify the electronic properties of two-dimensional SnP_3_. The interaction mechanism between the dopants and atoms nearby is also different from the type of doped atom. Both Sn_7_BP_24_ and Sn_7_NP_24_ systems are indirect bandgap semiconductors, while the Sn_7_AlP_24_, Sn_7_GaP_24_, Sn_7_PP_24,_ and Sn_7_AsP_24_ systems are metallic due to the contribution of doped atoms intersecting the Fermi level. For all substitutionally doped 2D SnP_3_ systems considered here, all metallic systems are nonmagnetic states. In addition, monolayer Sn_7_XP_24_ and Sn_8_P_23_Y may have long-range and local magnetic moments, respectively, because of the degree of hybridization between the dopant and its adjacent atoms. The results complement theoretical knowledge and reveal prospective applications of SnP_3_-based electrical nanodevices for the future.

## 1. Introduction

In 2004, single-atom-thick sheets of graphene were successfully prepared by mechanical stripping [1]. Since then, two-dimensional materials have attracted much attention because of their intrinsic excellent physical and chemical characteristics [2,3,4,5,6,7,8,9,10,11,12,13]. The exploration of the ultra-thin two-dimensional system is also inspired by the latest experimental progress of controlled fabrication of nanomaterials. However, monolayer 2D materials are always limited by their inherent electronic properties. For example, the zero band gap of graphene restricts it from being used in practical electrical devices [3]. For 2D MoS_2_ with the direct band gap of 1.79 eV, abundant internal defects seriously reduce the mobility of carriers [4,14]. On the other hand, the boron nitride monomolecule is not an ideal candidate material for optoelectronic components or electronic devices because of its wide band gap. Therefore, it is significant to find an effective solution to improve their electronic properties. To further improve the performance of 2D materials, there are many methods such as adsorption, doping, and constructing heterogeneous junctions by stacking different materials, which have shown excellent effects in different fields [15,16]. In this research, doping is used to improve the performance of SnP_3_, which has been demonstrated as an effective and simple approach to modify the magnetic and electrical characteristics of 2D materials [5,6,7,8,9,17,18,19]. By doping heteroatoms, carbon-based materials exhibit exceptional electrochemical properties in lithium-ion and sodium-ion batteries [20]. Atomic metal ion-doped graphene offers strong conductivity and stability to the materials, resulting in good pseudocapacitance performance [21]. By inserting small BN domains, the band gap of graphene could well be efficiently expanded near K as well as K′ points [7]. MoS_2_ is usually stated as natively n-type while dopants quench the n-type, allowing it to perform as a single-photon emitter [22]. Doping various elements with even or odd amounts of valence electrons causes an oscillating behavior in black phosphorene [23].

Very recently, a 2D monolayer SnP_3_, one of the representative layered group-IV-based triphosphide family materials, has been theoretically proposed by Sun et al. [24]. The cleavage energy of monolayer SnP_3_ [24] is close to the cleavage energy of graphene. The cleavage energy of monolayer SnP_3_ [22] is comparable to that of graphene [25]. In its monolayer form, SnP_3_ exhibits semiconducting behavior, with the indirect bandgap determined as 0.42 eV by the PBE function [26] and 0.72 eV by the HSE06 function [27]. Furthermore, with a higher affinity to the NH_3_, NO, and NO_2_, the SnP_3_ monolayer has been described as a promising sensing material for tiny gas molecules [28,29]. Because of its high areal capacity and good stability, SnP_3_ is also widely employed in Na- and Li-ion batteries [30,31]. SnP_3_ has also been predicted with a high light absorption coefficient and carrier mobility [32] and has been found as an excellent p-type thermoelectric material [33]. With ultra-low thermal conductivity and high thermoelectric performance, monolayer SnP_3_ is considered as the potential candidate for thermoelectric materials [34]. The thermoelectric effect, as an essential part of the solution to today’s energy challenges, by reducing adverse effects on the environment, makes the application of SnP_3_ more extensive [35].

We have performed systematic investigations of the substitutionally doped SnP_3_ monolayer. The structural characteristics (including the optimized geometries, lattice constants, distortion) and electronic properties (including the energy band structures, density of states, and the charge densities) are systematically discussed. The doping atoms replaced Sn, including group III (B, Al, Ga) and group V (N, P, As), while dopants substituted for P are from group IV (C, Si, Ge) and group VI (O, S, Se). The electronic properties of doped 2D systems are strongly affected by the number of outer electrons in the dopants and the doping sites. Since each layer of bulk SnP_3_ is mainly connected by weak van der Waals forces, our study has guiding significance for understanding bulk SnP_3_ and also provides direction for future SnP_3_-based nanoscale applications.

## 2. Computational Details

The Vienna ab initio simulation software (VASP) is performed to do the first principle calculations, which are based on the functional theory (DFT) of plane wave density and the projector augmented wave (PAW) approach [36,37]. In the Perdew-Burke-Ernzerhof (PBE) parameterization, the generalized gradient approximation (GGA) was used to describe the exchange-correlation interactions [38]. PBE functionals are related to semi-local functionals where the exchange-correlation energy is given by a function that depends on the electron density and the derivative of the electron density at each point of space. At the same time, SnP_3_ material is a layered structure with van der Waals (vdW)forces binding between layers. Since the DFT-GGA calculation lacks a prediction of the interlayer spacing of layer structures, we also take the van der Waals interaction (vdW) into consideration [39] by inserting a semi-empirical dispersion potential to the usual Kohn-Sham DFT energy (PBE-D2 computations). The plane wave function is used to create the electronic wave function, and the Monkhorst–Pack unique K-point sampling method is used to integrate the Brillouin zone (BZ) [40]. The vdW-D2 correction by Grimme is used to describe the interaction between SnP_3_ and dopant atoms over large distances. To eliminate the artificial contact between the neighboring periodic sheets, a 2 × 2 × 1 supercell with a vacuum layer of 20 Å in ‘z’ axis is utilized. A total of 500 eV was set as cut-off energy of the plane wave expansion. The Brillouin zone sampling was carried out using a 13 × 13 × 1 Γ-centered Monkhorst–Pack K-point mesh. All structures were completely relaxed until the total change in energy between two self-consistent steps was smaller than 1×10^−4^ eV.

## 3. Result and Discussion

**Figure 1:**
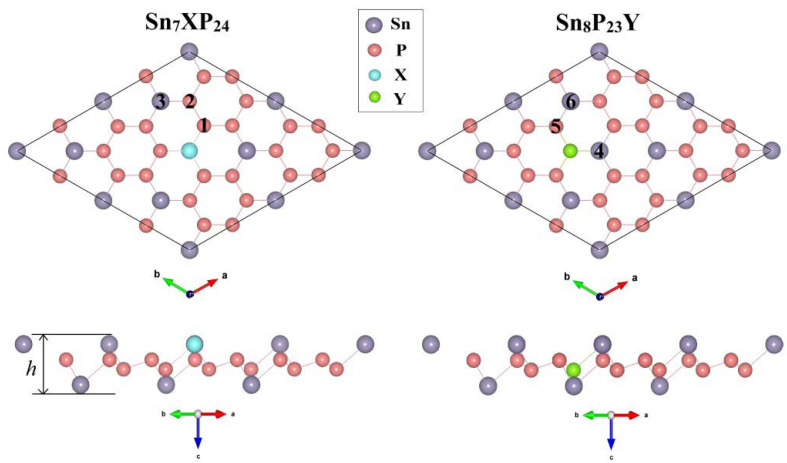
Top and side views of 2 × 2 substitutionally doped SnP_3_ supercell.

We established a 1 × 1 SnP_3_ cell (contains eight atoms) to determine the basic structural parameters of the 2D SnP_3_ system. After relaxation, the optimal lattice constant of the SnP_3_ supercell is 7.17 Å, which agrees well with the existing theoretical calculations [24,27,28]. The system was modeled based on a 2×2 SnP_3_ supercell (containing 32 atoms) for substitutionally doped SnP_3_ (shown in Figure 1) to minimize the influence of electrons states neighboring atoms. We first removed one Sn (or P) atom from the 2 × 2 SnP_3_ supercell and then replaced it with the one doped atom, which is labeled as X (or Y). The substitutionally doped SnP_3_ system is labeled as Sn_7_XP_24_ (or Sn_8_P_23_Y). Since the number of valence electrons in dopants has a great impact on the electronic characteristics of low dimensional materials, we select atom B, Al, and Ga from group III and atom N, P, As from group V as dopants for Sn atoms due to the one more or one less electron than Sn atoms. Similarly, we choose the atoms in group IV (C, Si, Ge), which has one less electron than P, and group VI (O, S, Se), which has one more electron as dopants for the P atom. The substitutional atoms are selected from the two adjacent groups of the Sn or P atom, each of which differs from the substituted atom by one outer electron.

After full relaxation, the lattice constant of 2 × 2 SnP_3_ is 14.32 Å. Table 1 displays the parameters of the Sn_7_XP_24_ and Sn_8_P_23_Y systems in which doped atoms have been replaced. We can see that the lattice constants of the doped systems are not much different from that before doping. Within the same group, the lattice constants rise as the atomic number increases. This is owed to the influence of the ionic radius’s effect upon the length of bonds. Although the insertion of doping atoms breaks the symmetry of the defect-free SnP_3_, the structure of the doping system typically retains the original hexagonal frame structure.

To evaluate the stability and feasibility of substitutionally doped SnP_3_ monolayer, the formation energy (*E_f_*) of the system is given as follows:

(1) $$E_{f}=E_{total}+E_{replaced}-E_{pristine}-E_{D}$$

where $E_{total\;}$ stands for the total energy of the SnP_3_ monolayer after doping (Sn_7_XP_24_ or Sn_8_P_23_Y). $E_{pristine}$ denotes the total energy of the pure Sn_8_P_24_. The chemical potentials of single X (or Y) and Sn (or P) atoms are symbolized by $E_{D}$ and $E_{replaced}$, respectively. With the exception of Sn_7_BP_24_ and Sn_8_P_23_C, the formation energies of all of these doping systems are quite low. Particularly, the formation energies of Sn_7_BP_24_, Sn_7_PP_24_, Sn_7_AsP_24_, Sn_8_P_23_C, Sn_8_P_23_Si, and Sn_8_P_23_O systems are negative. The positive or negative formation energy can reflect the stability of the systems. The results implied that the substitutional doping processes of these systems are stable and exothermal. The lengths of bond P-X/P-Y and bond Sn-P/Sn-Y are listed in Table 1 to study the structural deformation in the Sn_7_XP_24_ or Sn_8_P_23_Y systems. In comparison to the length of the Sn-P bond (2.71 Å) in pristine monolayer SnP_3_, the d has pronounced change. Although the doped atoms from the same main group have similar electronic configurations, the larger ionic radius could promote sp^3^ hybridization. For the doped atoms in the same main group, the atomic radius increase with the atomic number. This would explain why P-X/P-Y and Sn-Y bond lengths increase as the number of protons increases. However, sheet thickness did not vary much because the configuration distortions generated by a single dopant atom are highly localized.

**Figure 2:**
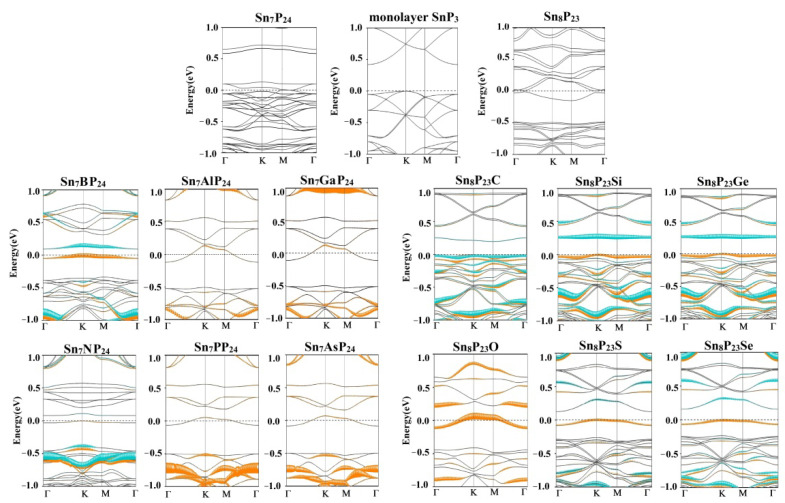
Electronic band structures of the substitutionally doped Sn_7_XP_24_ and Sn_8_P_23_Y system. The Fermi level locates at zero.

Figure 2 illustrates the substitutionally doped 2D SnP_3_ band structures obtained by the PBE-D2, the band gap values of different systems are shown in Table 1. The orange and blue dots represent the contributions of dopant atoms in spin-up and spin-down states, respectively. As presented in Figure 2, the pure SnP_3_ is a semiconductor with an indirect band gap of 0.42 eV, which agrees well with previous results [28,34,41]. The valence band maximum (VBM) is located at K-point, whereas the conduction band minimum (CBM) is found at Γ-point. When one Sn or P atom is removed from the 2 × 2 SnP_3_ monolayer, the system transforms into a metal. All B- and N-doped Sn_7_XP_24_ systems are indirect bandgap semiconductors. The band gaps of Sn_7_BP_24_ and Sn_7_NP_24_ are 0.08 and 0.09 eV, respectively. The band configurations of them are quite similar to the pristine monolayer SnP_3_. While the Sn_7_AlP_24_, Sn_7_GaP_24_, Sn_7_PP_24,_ and Sn_7_AsP_24_ systems are metallic due to the contribution of doped atoms intersecting the Fermi level. This means the Al-, Ga-, P-, and As-doping of Sn atom in SnP_3_ could increase its conductivity. In the case of substitutional doping of the P atom in SnP_3_, however, only the Sn_8_P_23_O system appears to be metallic. For systems with dopants from group IV (C, Si, Ge) and group VI (S, Se), the doped Sn_8_P_23_Y systems could retain semiconducting properties. In our research, the contributions of dopants are mainly around the Fermi level. Thus, for all doped semiconductor systems, the addition of doped atoms reduces the band gap value. Compared with the P-vacancy system, the Fermi level moves up in Sn_8_P_23_O, Sn_8_P_23_S, and Sn_8_P_23_Se systems. Among Sn_7_XP_24_ systems, the doped atoms contribute significantly near the Fermi level for the magnetic Sn_7_BP_24_ and Sn_7_NP_24_ systems, while for Al-, Ga-, P- and As-doped systems, the dopants contribute more in deep level. Within each main group, the band gap value of the substitutionally doped SnP_3_ also increases with the increasing atomic number (shown in Table 1).

**Figure 3:**
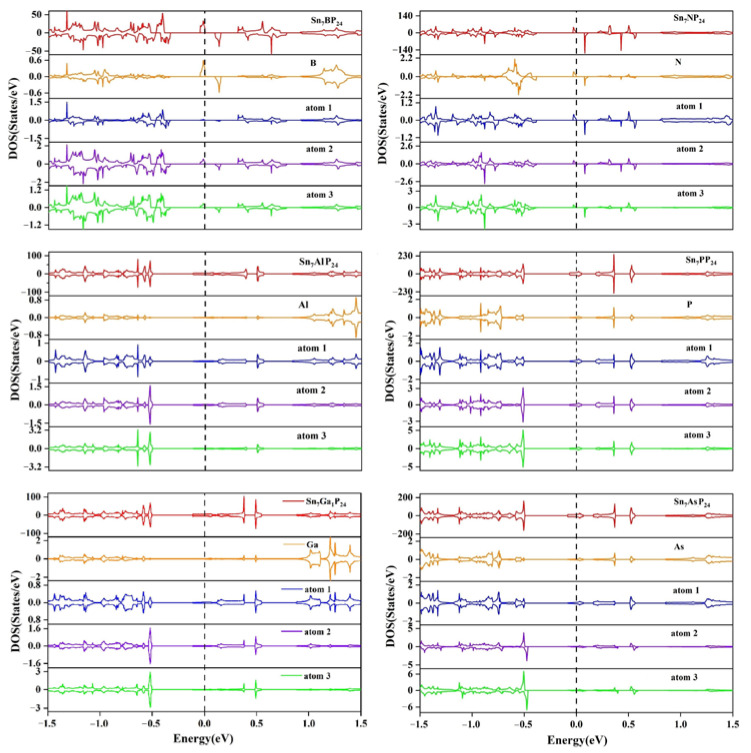
Total and partial densities of states of the substitutionally doped Sn_7_XP_24_ system.

**Figure 4:**
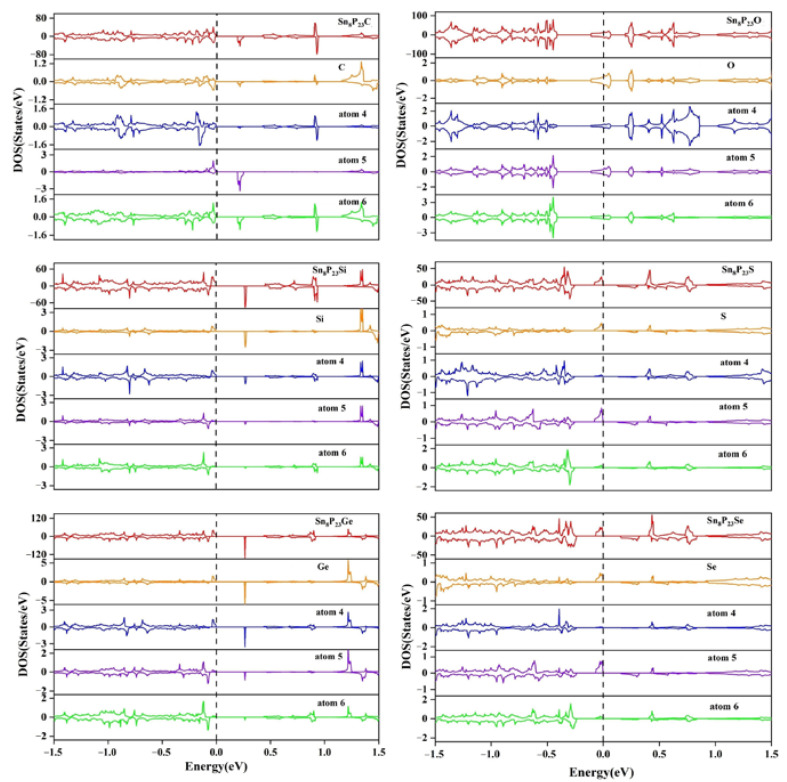
Total and partial densities of states of the substitutionally doped Sn_8_P_23_Y system.

To illustrate the intrinsic mechanism of the substitutionally doped system, the densities of states of the substitutionally doped Sn_7_XP_24_ and Sn_8_P_23_Y systems are presented in Figure 3 and Figure 4, respectively. The partial density of states (PDOS) of the dopant atom and its adjacent atoms is also displayed in addition to the total density of states (TDOS). The locations of these atoms have been marked in Figure 1. As can be found in the PDOS, near the Fermi level, the impurity atom has a larger contribution to the doped system. For substitutional doping on the Sn site of SnP_3_ monolayer, only the Sn_7_BP_24_ and Sn_7_NP_24_ systems are semiconductors. We can see that the spin-up (fully occupied) and spin-down (unoccupied) states are totally different near the Fermi level. The unevenness occupation between spin-up and spin-down leads to magnetic moments in the doped system. However, among all considered Sn_8_P_23_Y systems, the occupation situations of the spin-up and spin-down states are very uneven except for Sn_8_P_23_O. For systems with unevenness DOS, the spin-up states are fully occupied, and spin-down states are unoccupied. For all Sn_7_XP_24_ systems considered here, no matter the SnP_3_ monolayer has magnetic moments or not, there is strong hybridization between the X atom and its nearest (atom 1) and second nearest neighbor (atom 2) P atoms. What is more, the same degrees of hybridization are found between the X atom and its third nearest Sn atoms (atom 3). This would lead to a long-range magnetic moment in the systems. However, for the substitutionally doped Sn_8_P_23_Y systems, there is only strong hybridization between the Y atom and its nearest (atom 4) Sn atoms. On the other hand, the Sn_7_AlP_24_, Sn_7_GaP_24_, Sn_7_PP_24,_ and Sn_7_AsP_24_ systems are found to be metallic. Different from the substitutional doping on the Sn site, the semiconducting characteristics of the pure SnP_3_ monolayer are retained in the majority of Sn_8_P_23_Y systems. All group IV atoms-doped Sn_8_P_23_Y systems are semiconductors. The lowest unoccupied states fell below the Fermi level after substitutional doping of the O atom on the Sn site, showing n-type doping of the SnP_3_ monolayer.

**Figure 5:**
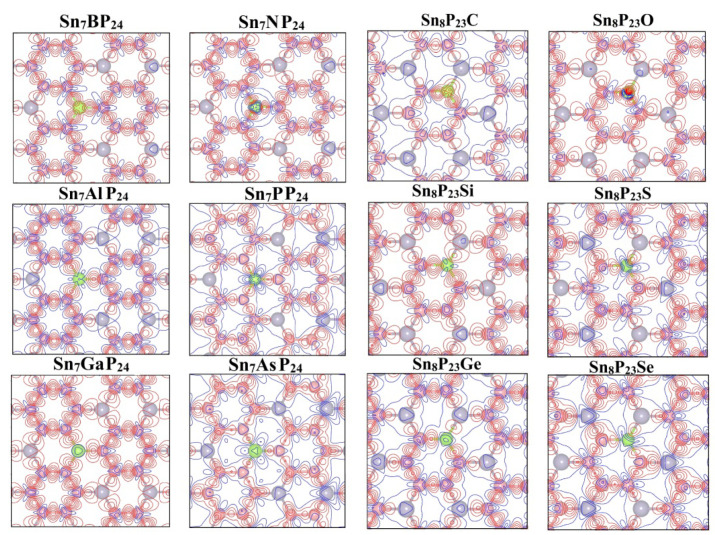
Deformation charge densities of the substitutionally doped Sn_7_XP_24_ and Sn_8_P_23_Y system.

To understand the mechanisms inherent in these low dimensional structures, which are necessary for the dynamics of material formation [42], the deformation charge density (Δ*ρ*) is also discussed in this work. The density of deformation charge is described as:

(2) $$∆\rho \left(r\right)=\rho \left(r\right)-\underset{\mu }{\sum }\rho_{atom}\left(r-R_{\mu }\right)$$

where the ρ(r) stands for the total charge density of the system and the $\underset{\mu }{\sum }\rho_{\mathrm{atom}}\left(r-R_{\mu }\right)$ denotes the superposition of atomic charge densities. The extent of charge transfer between the dopant and the monolayer SnP_3_ could be measured through ρ(r). The deformation charge density of planes slicing across the dopant atoms is shown in Figure 5. The electron accumulation (∆ρ>0) and electron depletion (∆ρ<0) are depicted by red and blue lines, respectively. For the Sn_7_BP_24_ and Sn_7_NP_24_ systems, there is obvious charge transfer from adjacent P atoms to dopant atoms. On the other hand, the electrons are apparently converging around the dopant atoms Sn_8_P_23_O and Sn_8_P_23_C systems. For doped atoms in the same main group, the properties of the bonds between dopant and its nearby atoms change with the atomic number of doped atoms. With the increase in dopants’ proton, the ionic component decreases, and the covalent component increases. At the same time, the electron transfer between atoms and the electron-accepting ability decreases as the atomic number increases. It can be explained by that electron-accepting ability decreases as the atomic number increases. Due to the strongest electron-accepting ability of the O atom, among all the substitutionally doped systems studied here, the most obvious electron transfer is found in the Sn_8_P_23_O system. Furthermore, when the covalent bonding ratio increases, the P-X and P-Y bond lengths of the substitutionally doped SnP_3_ monolayer also become longer. Thus, the distribution of electrons in the systems is significantly affected by the introduction of doping atoms.

**Figure 6:**
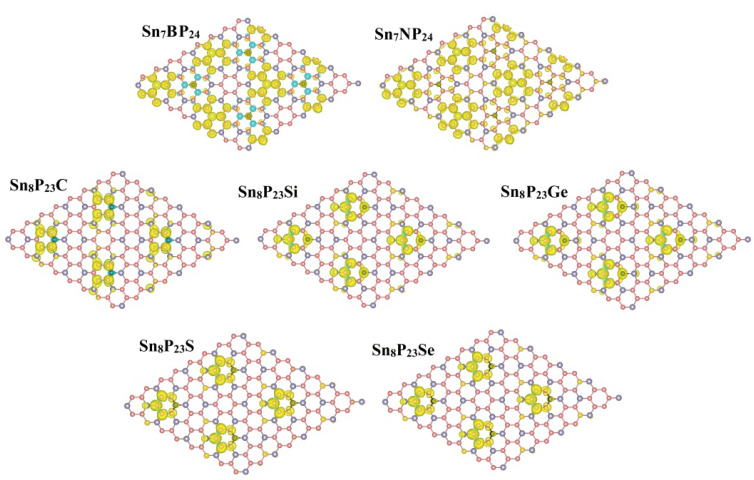
The spin densities of magnetic substitutionally doped Sn_7_XP_24_ and Sn_8_P_23_Y system.

For a long time, the magnetism of 2D materials has attracted a great deal of academic attention. The difference in charge density between the spin-up (ρ↑) and spin-down states (ρ↓) of substitutionally doped 2D SnP_3_ systems are presented in Figure 6. Table 1 also shows the total magnetic moments of the doped systems determined with PBE-D2. For substitutionally doped 2D SnP_3_ systems considered here, all metallic systems are nonmagnetic states. As mentioned above in Table 1, the magnetic moments of about 1.00 µ_B_ in doped systems were caused by the dopant atoms in the SnP_3_ monolayer. We found that the long-range magnetic moments can be found in the Sn_7_BP_24_ and Sn_7_NP_24_ systems, resulting from the strong hybridization between the B (or N) atom and its nearest P atoms, second nearest P atoms, and its third nearest Sn atoms. However, the magnetic Sn_8_P_23_Y (Y = C, Si, Ge, S, Se) systems only show local magnetic moments through the substitutional doping process. Thus, the 2D indirect bandgap SnP_3_ monolayer can attain local or long-range magnetic moments through the substitutional doping, resulting from the different interaction mechanisms between the doping atom and its neighboring atoms, which make the SnP_3_ sheet a promising material for nanoscale technological application in spintronic.

## 4. Conclusions

In summary, we investigated the electronic characteristics of substitutionally doped 2D SnP_3_ systems using first-principles calculations. One Sn atom is replaced with a dopant from group III (B, Al, Ga) and group V (N, P, As), while the P atom is replaced with an atom from group IV (C, Si, Ge) and group VI (O, S, Se). The substitutional atoms are selected from the two adjacent groups of the Sn or P atom due to one less or more electron. Our results show that the impurity atoms have a significant impact on the electronic characteristics of whole substitutionally doped systems. SnP_3_ monolayers can exhibit metallic or semiconducting behavior depending on the dopant type. All semiconductive systems are indirect band gap semiconductors with the introduced magnetic moments of about 1.00 µ_B_. Within the same main group, the band gap and structural parameters increase as the atomic number of dopant rise. In addition, monolayer SnP_3_ may have local or long-range magnetic moments because of the degree of hybridization between the dopant and its adjacent atoms. This allows SnP_3_ monolayers to be widely used at the nanoscale in the field of spintronics. Since the doped atoms have a significant impact on the properties of the material, SnP_3_ is suitable for various functionalization processes.

## Figures and Tables

**Table 1:** Calculated Structural Geometry of substitutionally doped Sn_7_XP_24_ and Sn_8_P_23_Y system, including the lattice constants (*a = b*), P-X/P-Y bond length (*d*_1_), Sn-P/Sn-Y bond length (*d*_2_), band gap (*Eg*), SnP_3_ thickness (*h*) and total magnetic moment of the system in units of Bohr magneton (M) calculated by using PBE-D2.

System	*a = b* (Å)	E_f_ (eV)	*d*_1_ (Å)	*d*_2_ (Å)	*h* (Å)	E_g_ (eV)	M (μ_B_)
SnP_3_	14.32	/	2.71	2.71	2.81	0.42	0.00
Sn_7_BP_24_	14.30	−5.662	1.93	2.71	2.72	0.09	1.00
Sn_7_AlP_24_	14.32	−3.918	2.32	2.71	2.85	/	0.00
Sn_7_GaP_24_	14.32	0.592	2.33	2.71	2.85	/	0.00
Sn_7_NP_24_	14.25	0.333	1.86	2.71	2.74	0.08	1.00
Sn_7_PP_24_	14.25	−1.184	2.32	2.71	2.76	/	0.00
Sn_7_AsP_24_	14.26	−0.699	2.44	2.71	2.77	/	0.00
Sn_8_P_23_C	14.22	−6.609	1.74	2.35	2.72	0.21	1.00
Sn_8_P_23_Si	14.37	−0.206	2.19	2.67	2.69	0.26	1.00
Sn_8_P_23_Ge	14.41	0.579	2.28	2.72	2.65	0.27	1.00
Sn_8_P_23_O	14.20	−0.636	1.75	2.69	2.80	/	0.00
Sn_8_P_23_S	14.29	0.756	2.20	2.74	2.75	0.12	1.00
Sn_8_P_23_Se	14.37	0.661	2.35	2.83	2.72	0.16	1.00

## Data Availability

The data presented in this study are available on request from the corresponding author.
